# Energy storage-enabled fractional-order virtual synchronous generator for DC-link voltage regulation in DC microgrid under load and renewable disturbances

**DOI:** 10.1038/s41598-026-45850-1

**Published:** 2026-04-13

**Authors:** Abualkasim Bakeer, Shafquat Hussain, Andrii Chub, Hossam S. Salama, Gaber Magdy

**Affiliations:** 1 Electrical Engineering Department, Faculty of Engineering, Aswan University, Aswan, 81542 Egypt; 2 Department of Electrical, Electronic, Telecommunications Engineering and Naval Architecture, University of Genova, 16145 Genova, Italy; 3 Department of Electrical Power Engineering and Mechatronics, Tallinn University of Technology, 19086 Tallinn, Estonia; 4 Faculty of Engineering, King Salman International University, El-Tor, South Sinai 46511 Egypt; 5 Electrical Engineering Department, Faculty of Energy Engineering, Aswan University, Aswan, 81528 Egypt

**Keywords:** Virtual synchronous generator (VSG), Droop control, Fractional-order control (FOC), DC microgrid, Renewable energy sources (RESs), Energy science and technology, Engineering

## Abstract

This paper presents an advanced control strategy for energy storage–integrated DC microgrids to improve DC-link voltage stability and transient performance under operating uncertainties. The proposed approach emulates virtual synchronous generator (VSG) behavior through coordinated primary and secondary control layers, where virtual inertia and damping are introduced to enhance dynamic response. To improve robustness against load changes and renewable-generation fluctuations, a fractional-order control law is designed to regulate the rate of change of the DC-link voltage (RoCoV), offering additional tuning flexibility compared with conventional integer-order VSG schemes. Controller parameters are optimally selected using a metaheuristic optimization method to further enhance regulation quality and efficiency. Extensive simulations benchmark the proposed fractional-order VSG against representative integer-order, derivative-based VSG controllers. The results demonstrate improved DC-link voltage regulation during load variations and renewable power disturbances, with the proposed method achieving up to 80% reduction in DC-link voltage overshoot in some test scenarios compared with the baseline controller.

## Introduction

Recently, the growing adoption of DC sources and loads has drawn significant attention to DC microgrids. These systems integrate distributed energy resources (DERs), including renewable power generators, energy storage devices, and local DC loads, into unified DC networks. This integration enables improved energy management, enhanced efficiency, and greater flexibility in accommodating diverse power sources and demand profiles^1^. On the other hand, the intermittent nature of renewable energy sources (RESs) and sudden load variations cause fluctuations in the DC bus voltage^2^. Moreover, the insufficient inherent inertia of RESs, such as photovoltaic (PV) panels and wind turbines (WTs), primarily generates these fluctuations. This issue becomes particularly critical during islanded operation, where DC microgrid stability is more vulnerable^3,4^. In such scenarios, the control system becomes sluggish and more complex. This increases the DC bus voltage’s susceptibility to disturbances, potentially leading load shedding or power outages. Unlike AC systems, where synchronous rotating machines naturally provide inertia, DC systems inherently possess much lower inertia. This is due to the typically small energy storage components and the limited capacitance of the DC link capacitor, which are constrained by physical size^5,6^. In addition, power electronic converters act as static elements and do not contribute to the inherent inertia of the system. Therefore, enhancing the inertia of power electronics converters is essential for effective DC bus voltage regulation and overall microgrid stability^7^.

As the share of inverter-based RESs grows, this inertia deficit becomes more pronounced because PV and wind units connect through power electronics and lack the rotating mass of synchronous machines^8^. To address these challenges, researchers have introduced the concept of a virtual synchronous generator (VSG)^9^. VSGs emulate the inertial and damping behaviour of conventional SGs by implementing a virtual rotational mass. This emulated inertia provides dynamic support, compensating for the inertia deficit from inverter-based RES integration. Beyond inertia replication, VSGs mimic SG voltage regulation through integrated primary and secondary virtual control loops. Unlike traditional SGs, which contribute to system dynamics inherently through physical properties, VSGs restore essential dynamic characteristics in renewable-dominated microgrids^9^.

Therefore, grid-connected converters with energy storage systems (ESS) must have inertia emulation capabilities. Enhanced inertia emulation can be achieved by operating multiple converters in parallel rather than relying on a single device. In such parallel configurations, involving a converter in primary regulation increases the primary reserve and helps mitigate voltage deviations following power transients. As demonstrated in Serban and Ion^10^, droop control combined with a virtual inertia control (VIC) has been applied to both DC–DC and DC–AC converters. While this approach improves the system’s dynamic response, it has been observed that inertia emulation via droop control alone is insufficient for eliminating steady-state errors.

Various VIC techniques for voltage source inverters have been proposed in the literature to emulate the inertial behavior of SGs. These include the VSG^6,7,11–13^, virtual synchronous machine (VSM)^10,14^, synchronverter^15^, virtual synchronous control^16^, and synchronous generator emulation control^17^, among others. VIC techniques have been implemented to regulate DC bus voltage for DC microgrids, as demonstrated in^18,19^. A VIC-based emulation method is formulated through the equivalence to a VSM and is proposed in Wang et al.^18^ for DC bus regulation. Similarly, the authors of Unamuno and Barrena^20^ extend this concept to control bidirectional grid-connected inverters, enabling both DC bus voltage regulation and inertia emulation. In Haddadi et al.^21^, a VIC strategy is integrated with maximum power point tracking (MPPT) for a wind-battery-based isolated DC microgrid. In this setup, a voltage source converter interfaced with a permanent magnet synchronous generator (PMSG) emulates inertia by linking variations in DC bus voltage to the regulation of PMSG speed. In another study^22^, an adaptive VIC based on active power control is designed for a PV-battery isolated DC microgrid to control a DC–DC converter connected to a solar PV system. This method enables inertial response, enhances damping in the DC microgrid, and supports dynamic power sharing among PV arrays, effectively reducing the dependency on high-power energy storage devices such as supercapacitors. However, a limitation of this approach is that inertia emulation is unavailable during periods when RESs are inactive, thereby limiting its effectiveness under low generation. Table 1 summarizes representative control strategies for DC microgrids, outlining their operating principles along with key advantages and limitations.

**Table 1:** Comparison of control approaches in literature.

References	Control	Pros	Cons
Alturki et al.^23^	PI	Simple and easy to implement.	Fixed gains can’t adapt to irradiance/temperature changes, so steady-state error increases.
Muralikumar et al.^24^	Fuzzy Logic	Handles system uncertainty gracefully.	Rule-based tuning is subjective, leading to suboptimal membership functions.
Wu et al.^25^	VIC control strategy	Provides virtual inertia to support frequency stability.	Phasor oscillations occur if the VIC parameters aren’t matched to the grid strength.
Unamuno et al.^26^	Virtual capacitor control	Actively shapes output impedance to damp voltage deviations.	Reliance on passive elements can slow dynamic response.
Jin et al.^27^	Admittance-based droop VIC	Modulates virtual admittance to constrain impedance swing.	Potential resonance peaks if the admittance filters are not carefully designed.
Proposed	Full FOVSG	Provides tunable virtual inertia and damping for enhanced voltage stability.	Moderate computational burden.

To control the DC bus voltage in a DC microgrid, a virtual capacitor control technique for DC–DC converters is proposed in Samanta et al.^28^. Additionally, the concept of a virtual DC machine is introduced in Li et al.^29^ to replicate the behaviour of a DC machine for controlling a bidirectional DC–DC converter within the microgrid. A VSG with droop control is proposed in Liu et al.^14^ for grid-connected inverters interfaced with a DC microgrid for seamless integration of the AC grid with inertia emulation. Recently, fractional-order (FO) controllers have emerged as powerful tools for enhancing control system performance in uncertain and dynamically changing environments. When compared to traditional PI/PID controllers, they provide more flexibility and improving options. The FO controllers have continuously outperformed conventional controllers in applications including renewable integration, load frequency control (LFC), and automatic generation control (AGC). The tuning of FO controllers is more flexible, enabling more precise and adaptable control, especially beneficial for microgrid applications.

Recent studies have also highlighted the broader role of optimization and compensation techniques in improving distribution-system performance. For example, analytical and optimization-based methods have been reported for the optimal sizing and siting of Type-IV distributed generation in unbalanced distribution systems with the objective of power-loss minimization^30^. Related work has further considered reactive-power support using UPQC and particle swarm optimization for improved DG integration in unbalanced feeders^31^. In addition, heuristic approaches for optimal DSTATCOM placement have demonstrated the importance of advanced compensating devices for enhancing voltage profile and overall power quality^32^. These studies complement the present work by showing the growing importance of intelligent optimization and control strategies in modern power and energy systems.

More recent studies have further emphasized the growing role of advanced control design, fractional-order strategies, and optimization-assisted tuning in modern power and microgrid applications. For instance, recent work has reported cascade control formulations and optimization-based designs for improved load-frequency regulation in renewable-rich power systems^33,34^. Related studies have also explored resilient virtual-inertia-supported control under communication constraints, highlighting the importance of robustness-oriented inertia emulation in low-inertia hybrid systems^35^. In addition, fractional-order AGC designs optimized through advanced metaheuristic techniques have demonstrated the value of intelligent tuning for enhancing dynamic regulation under high renewable penetration^36^. These developments further motivate the present study and confirm the relevance of combining virtual inertia concepts, fractional-order control, and optimization-based parameter tuning in microgrid control design.

Most earlier inertia-emulation systems in DC microgrids use integer-order derivatives to simulate the SG inertia. This limits the controller’s ability to shape the transient response across frequencies by confining it to a small range of phase and gain profiles. Designers must therefore make difficult trade-offs between noise amplification and quick disturbance rejection, and regular retuning is required as operating conditions alter. Furthermore, a large portion of the previous research focuses on virtual inertia and, occasionally, virtual damping, ignoring other SG aspects essential for DC microgrids, such as secondary control for DC-link voltage restoration and coordinated primary control for power sharing. These layers are necessary for the DC-bus regulator to have a coherent multi-timescale structure, which is troublesome when dealing with parallel converter operation, intermittent RES inputs, and battery state-of-charge limitations.

To address these limitations, we propose a fractional-order virtual synchronous generator (FOVSG) that generalizes inertia and damping to fractional orders. This improves phase margin and robustness to operating-point changes and parameter drift by expanding the tuning space and allowing for frequency-selective damping and apparent inertia tailoring. With a primary layer to coordinate numerous converters, a secondary layer to eliminate steady-state DC-link voltage error, and rapid inner current/voltage loops for converter control, the FOVSG is integrated into a hierarchical design. By doing so, the controller preserves close coordination between RES interfaces and storage and replicates important synchronous-generator behaviours beyond inertia alone. In practice, the fractional orders attenuate high-frequency noise while preserving rapid support during disturbances, yielding smaller voltage excursions, faster restoration, and scalable performance in renewable-dominated DC microgrids. This makes it well suited to hybrid/renewable DC microgrids where operating conditions change frequently. However, although prior studies have investigated fractional-order control strategies and virtual inertia/VSG-based control schemes, the combination of these two concepts for DC-link voltage regulation in DC microgrids remains insufficiently addressed. In particular, existing VSG-based DC microgrid methods mainly rely on integer-order inertia-emulation mechanisms, while existing fractional-order approaches are generally not formulated within a DC-VSG framework for RoCoV-based DC-link regulation. Motivated by this gap, this paper proposes a fractional-order virtual synchronous generator (FOVSG) strategy in which the inertial channel of the DC-VSG is generalized through a fractional-order RoCoV control law, aiming to enhance transient voltage regulation and robustness under load and renewable power disturbances. The main contributions of the paper are:

(i) A comprehensive emulation of the SG characteristics is proposed for DC microgrid stability, incorporating primary and secondary control loops along with virtual inertia and damping mechanisms.
(ii) A FOC approach is introduced for regulating the rate of change of the DC-link voltage (RoCoV), offering greater flexibility and precision than conventional integer-order methods.
(iii) The parameters of the proposed control are optimally tuned using a metaheuristic algorithm, resulting in enhanced system dynamics and steady-state performance.
(iv) A detailed simulation-based comparison is conducted against conventional dual-loop control (without VSG) and traditional VSG methods, demonstrating the superior effectiveness of the proposed approach in maintaining DC-link voltage stability.

The paper is organized in five sections: Section “Configuration of the studied system” describes the configuration of the proposed DC microgrid system. Section “Conventional virtual synchronous generator control in DC microgrids” discusses the conventional VSG control in DC microgrid. Section “Proposed fractional-order virtual synchronous generator (FOVSG)” details the proposed FOVSG control strategy. Section “Results and discussion” presents simulation results evaluating DC-link voltage regulation performance under three control strategies across three distinct scenarios. Finally, Section “Conclusion” concludes the paper by summarizing key findings and outlining potential directions for future research.

## Configuration of the studied system

**Fig. 1:**
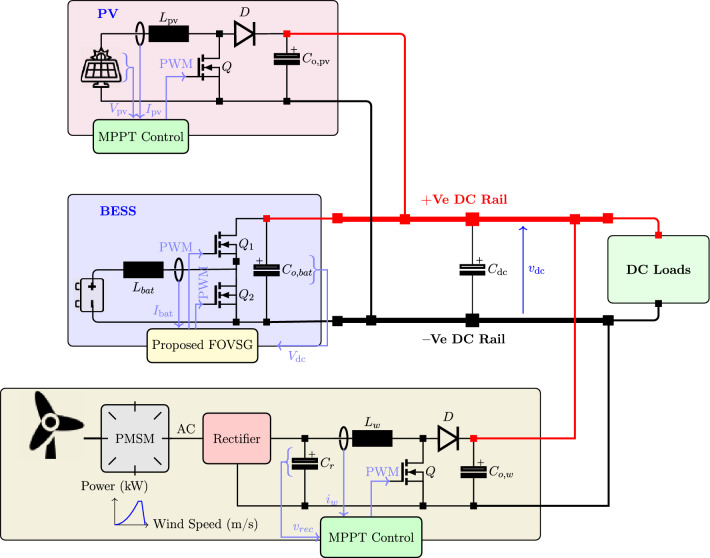
Detailed power conversion units for the studied DC microgrid.

The microgrid in Fig. 1 is built around a common DC bus with four parallel branches: (i) a bidirectional DC–DC converter (BDDC) interfacing the battery, (ii) a DC–DC boost converter for the PV array, (iii) a DC–DC boost converter for the wind unit, and (iv) a DC load. The BDDC regulates the DC-bus voltage. A perturb-and-observe (P&O) MPPT algorithm drives the DC–DC boost stage connected to the PMSG in the wind–battery subsystem. Mechanical power is converted to three-phase AC by the PMSG, then conditioned by the machine-side converters: an AC–DC rectifier followed by a DC–DC stage. These converters deliver a regulated DC output to the DC-link capacitor, allowing the proposed DC microgrid to integrate multiple RES and storage units while keeping the bus voltage stable and operation efficient.

The PV array converts sunlight directly into DC power. An MPPT controller extracts the maximum available power, and a DC–DC boost converter raises the PV voltage to the DC-link level. At the DC link, the PV and wind sides present the same regulated voltage. The DC link ties both renewable sources to the battery and the DC load, and its capacitor helps keep the voltage steady despite changes in generation. Energy storage and loads are also connected to the DC bus. The battery energy storage system (BESS) interfaces through a BDDC, which manages charging and discharging based on load demand and state of charge: when charging, power flows from the DC bus to the battery; during discharge, the battery feeds the DC bus. The BDDC uses a double-loop controller; in this work we implement it with no VSG, with conventional VSG, and with the proposed FOVSG to assess performance.

When wind or solar power is unavailable or highly intermittent, the effectiveness of virtual inertia contributed through the RES converters is limited^37^. This underscores the need to emulate inertia within the storage interface. Relying on a single converter also reduces robustness. Operating multiple converters in parallel enables coordinated primary regulation, increases the available reserve, and reduces voltage deviations during disturbances. In short, storage-integrated converters significantly strengthen system stability and dynamic response by providing improved inertia emulation. The following subsections detail the modelling, design, and control of the PV subsystem, the wind subsystem, and the BESS within the DC microgrid.

### Photovoltaic generation system

**Fig. 2:**
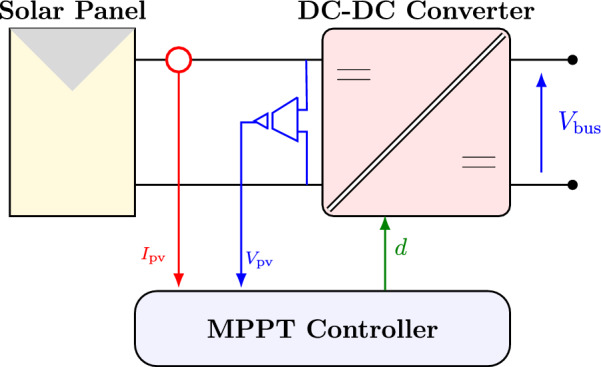
Schematic diagram of the PV system.

The PV array is interfaced to the DC bus through a DC–DC boost converter that raises the panel voltage $V_{\textrm{pv}}$ to the DC-link level required by the microgrid. Because the raw PV voltage is usually below the bus voltage and varies with irradiance and temperature, the boost stage both adapts the operating point and enables consistent energy extraction. The converter is regulated by a MPPT loop, as outlined in Fig. 2. To track the maximum power, we employ a perturb-and-observe MPPT scheme. The controller makes a small change to the operating point (equivalently, to $V_{\textrm{pv}}$ via the duty cycle), measures the resulting change in PV power, and then decides the next move: if the power increases, the controller continues in the same direction; if the power decreases, it reverses the step. Repeating this process drives the operating point toward the MPP and then keeps it near that point with small oscillations whose size is set by the step magnitude.

### Wind turbine generation system

Wind energy is converted into electrical energy through the mechanical rotation of turbine blades, which are driven by the wind’s kinetic energy. This rotation produces torque that drives a generator, typically generating three-phase AC electrical power. The AC–DC converter converts it into a DC voltage, and then a DC–DC converter supplies a regulated DC voltage to the required level of the DC-link voltage. The available power depends on wind speed; however, it is crucial to implement control mechanisms to limit power output at higher wind speeds, thereby preventing potential damage to the unit. An aerodynamic input torque is considered to analyse a wind turbine model within a microgrid, which serves as the driving force for the generator. The mechanical power of a wind turbine system can be described by the following equation^38^:

1 $$\begin{aligned} P_w = \frac{1}{2} C_p(\lambda , \beta ) \rho A V_w^3 \end{aligned}$$

where,

2 $$\begin{aligned} C_p = 0.5\left( \lambda -0.022 \beta ^2-5.6\right) e^{-0.17 \lambda } \end{aligned}$$

3 $$\begin{aligned} \lambda = \frac{\omega R}{V_w} \end{aligned}$$

Here, $\rho$ represents the air density (kg/m³), $C_p$ denotes the power coefficient, *A* is the rotor blade’s swept area (m²), $V_w$ is the average wind speed (m/s), and $\lambda$ is the tip speed ratio (TSR). Smaller wind turbines have fixed $\beta$; therefore, for this case, $C_p$ is a function of $\lambda$ alone. For every $V_w$, there is a different optimal $\lambda$. Thus, coupling a generator with variable speed drives enables maximum power extraction at different $V_w$. Theoretically, the maximum value of $C_p$, the power coefficient, is 0.593, or Betz’s coefficient.

The speed at which the turbine begins to rotate and generate power is called the cut-in speed. Conversely, the cut-out speed is the maximum wind speed at which the turbine can operate safely; beyond this speed, the risk of rotor damage becomes significantly high. Using the following expressions, the permanent magnet synchronous generator coupled to the wind turbine is modelled in a direct-quadrature (dq) synchronous frame^38^.

4 $$\begin{aligned} V_d = -R_s i_d - L_d \frac{d i_d}{d t} + L_q i_q \omega \end{aligned}$$

5 $$\begin{aligned} V_q = -R_s i_q - L_q \frac{d i_q}{d t} + L_d i_d \omega + \phi _m \omega \end{aligned}$$

The dq-axes of voltages, currents, and inductance are: $V_d$, $V_q$, $I_d$, $I_q$, $L_d$, and $L_q$, respectively. The stator resistance is denoted by $R_s$, while $\omega$ is the electrical angular speed. The PMSG establishes a magnetic flux linkage $\phi _m$; thus, the electromagnetic torque $T_e$ is computed by (6). Since the PMSG has a cylindrical rotor, $L_d=L_q$, then Eq. (6) simplifies to Eq. (7).

6 $$\begin{aligned} T_e= 1.5 p\left( \left( L_d - L_q\right) i_d i_q + \phi _m i_q\right) \end{aligned}$$

7 $$\begin{aligned} T_e = 1.5 p \phi _m i_q \end{aligned}$$

**Fig. 3:**
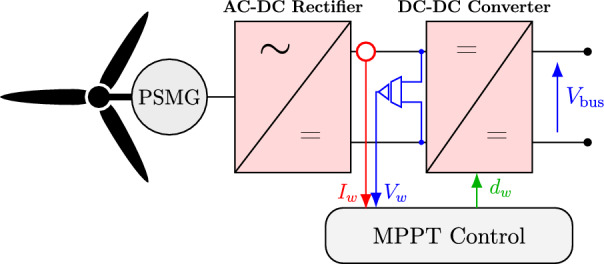
Schematic diagram of the wind turbine system.

At the output of the PMSG, an AC–DC converter is employed to convert the variable-frequency AC voltage generated by the PMSG into a stable DC voltage. This rectification stage is essential for interfacing the generator with the DC microgrid. However, the DC voltage obtained directly from the AC–DC converter may not match the desired DC-link voltage level required by the rest of the system. Therefore, a DC–DC converter is subsequently used to regulate and adjust this voltage to the standardised DC-link level as shown in Fig. 3.

### Battery energy storage system

**Fig. 4:**
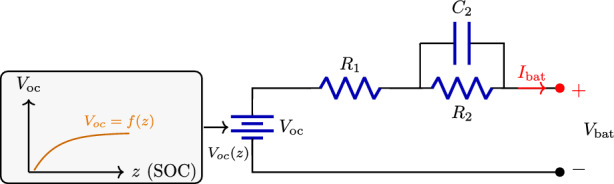
Equivalent circuit of a battery.

Energy can be stored by converting electrical energy into other forms, such as chemical or mechanical energy. Furthermore, ESSs can be classified into three main components: the central storage unit, the power transformation stage, and the control stage. After converting energy, it is stored in the central storage unit (e.g., batteries, flywheels). The power transformation stage acts as an interface between the central storage and the power system, allowing bidirectional energy transfer for charging and discharging operations. The control stage manages these operations by determining when and how much energy should be charged or discharged based on system conditions and demand. However, it is important to note that energy storage devices are not ideal energy sources, as they exhibit limitations such as energy losses, degradation over time, and efficiency constraints^39^. Despite these limitations, the BESS is widely applicable across all electrical power system generation, transmission, and distribution sectors, offering significant benefits to consumers. A simplified equivalent circuit of a battery is illustrated in Fig. 4.

**Fig. 5:**
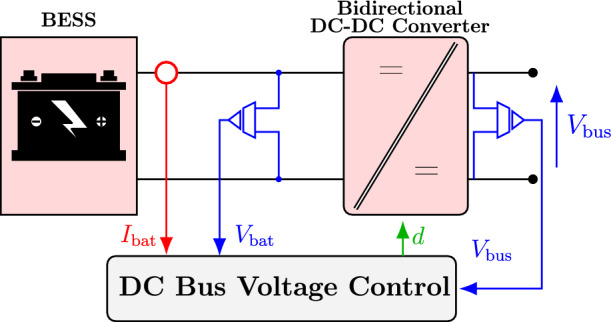
Control structure of BESS.

A bidirectional DC–DC converter connects a battery to the DC microgrid, as shown in Fig. 5. The solar PV and wind turbine systems are each interfaced through individual DC–DC boost converters, which are all connected to the common DC link of the microgrid. The equations for designing the power stage of the BDDC converter are:

8 $$\begin{aligned} L_{\textrm{bat}}=\frac{D_{\textrm{bat}}(V_{\textrm{bus}}-V_{\textrm{bat}})}{f_s\Delta I_{\textrm{bat}}} \end{aligned}$$

9 $$\begin{aligned} D_{\textrm{bat}}=1-\frac{V_{\textrm{bat}}}{V_{\textrm{bus}}} D_{\textrm{bat}}=\frac{V_{\textrm{bat}}}{V_{\textrm{bus}}} \end{aligned}$$

where the duty cycle of the BDDC is denoted by $D_{\textrm{bat}}$, $\Delta I_{\textrm{bat}}$ is the ripple in battery current, $f_s$ is the switching frequency, and $L_{\textrm{bat}}$ is the inductor of the BDDC. A suitable control algorithm protects the battery from overcharging and discharging. This paper uses a BDDC converter to balance the DC link voltage at 700 V and can handle high current during low-voltage operation. The battery voltage is represented by $V_{\textrm{bat}}$. The inductor of the BDDC is represented by $L_{\textrm{bat}}$, through which the charge or discharge current $I_{\textrm{bat}}$ will flow.

To control the charge and discharge of the BESS, the measurements of the DC-bus voltage ($V_{\textrm{dc}}$), state of charge (SOC) of the battery, and total power ($P_{\textrm{net}}$) of the DERs of the DC microgrid is necessary. The $P_{\textrm{net}}$ can be calculated as:

10 $$\begin{aligned} P_{\textrm{net}}=(P_{\textrm{pv}} + P_w)-P_L \end{aligned}$$

where, $P_{\textrm{pv}}$ represents the power generated by the solar PV system, $P_w$ denotes the power output from the wind turbine system, and $P_L$ is the power consumed by the load. In the case of ($P_{\textrm{net}}>0$), there is an excess amount of power coming from the RESs, which can also be used to charge the battery. Contrary to ($P_{\textrm{net}}<0$), it means there is not sufficient power from the RESs, so in this case, the battery will be discharged to supply the DC load. The control of the bidirectional DC–DC converter is based on a dual-loop structure. The outer loop is a voltage control loop, which operates more slowly, while the inner loop is a current control loop designed to be approximately ten times faster than the outer loop.

## Conventional virtual synchronous generator control in DC microgrids

**Fig. 6:**
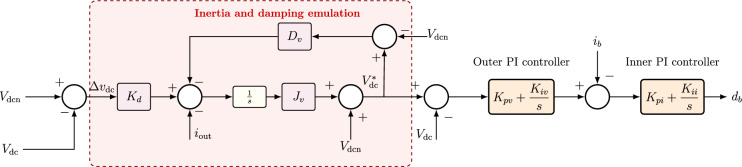
Conventional VSG control for DC microgrid.

Converter-dominated DC microgrids lack the inherent inertia and damping that are naturally provided by synchronous generators in conventional power systems. The virtual synchronous generator concept aims to replicate the electromechanical behavior of an SG by synthesizing a virtual rotor, virtual inertia, and virtual damping, in addition to voltage regulation loops that emulate excitation control^40^. A block representation of a conventional VSG applied to a DC microgrid is shown in Fig. 6.

### Synchronous generator swing equation and its interpretation

The dynamic response of a synchronous generator is governed by the swing equation

11 $$\begin{aligned} 2 J \frac{d^2 \delta }{dt^2} + D \frac{d \delta }{dt} = P_m - P_e , \end{aligned}$$

where $J$ is the rotational inertia constant of the machine, $D$ is the damping coefficient, $\delta$ is the electrical rotor angle with respect to a synchronously rotating reference frame, $P_m$ is the mechanical input power, and $P_e$ is the electrical output power exchanged with the network.

The first derivative of the rotor angle,

12 $$\begin{aligned} \omega (t) = \frac{d \delta }{dt} , \end{aligned}$$

is the electrical angular speed of the rotor. Substituting $\omega = d\delta /dt$ into (11) produces the commonly used form

13 $$\begin{aligned} 2 J \frac{d \omega }{dt} + D \omega = P_m - P_e . \end{aligned}$$

Equation (13) indicates that the inertia $J$ limits the instantaneous rate of change of speed, while the damping $D$ acts to dissipate oscillatory energy and restore equilibrium.

In a DC microgrid, no physical rotor is present. Instead, the DC bus voltage $V_{\textrm{dc}}$ assumes a role analogous to frequency in AC systems. A deviation of the DC bus voltage from its nominal value reflects an instantaneous power imbalance between generation, storage, and load. Therefore, it is desirable to reproduce a dynamic law analogous to (13), but in the DC voltage domain.

### Voltage deviation definition and virtual rotor power

Let the DC-link voltage deviation be defined as

14 $$\begin{aligned} \Delta V_{\textrm{dc}}(t) = V_{\textrm{dc}}^\star - V_{\textrm{dc}}(t), \qquad \dot{V}_{\textrm{dc}}(t) = \frac{d V_{\textrm{dc}}(t)}{dt} , \end{aligned}$$

where $V_{\textrm{dc}}^\star$ is the nominal DC bus voltage.

In conventional DC VSG implementations, a virtual rotor is synthesized, and its reference mechanical-like input power is formed as a function of $\Delta V_{\textrm{dc}}$. In the Laplace domain this reference is written as

15 $$\begin{aligned} P_{\textrm{ref}}(s) = \left( J_v s + D_v \right) \, \Delta V_{\textrm{dc}}(s), \end{aligned}$$

where $J_v$ and $D_v$ are the virtual inertia and virtual damping gains, respectively, and $s$ is the Laplace operator.

It can be noted that expression (15) consists of two components: the virtual inertia component, $J_v s \Delta V_{\textrm{dc}}(s)$, which is proportional to $d(\Delta V_{\textrm{dc}})/dt$ in the time domain, and therefore counteracts rapid changes in $V_{\textrm{dc}}$. The virtual damping component, $D_v \Delta V_{\textrm{dc}}(s)$, which introduces a proportional action that forces $V_{\textrm{dc}}$ toward $V_{\textrm{dc}}^\star$ and suppresses oscillations. Together, these terms emulate the left-hand side of (13), with the DC voltage deviation $\Delta V_{\textrm{dc}}$ acting as an analogue of rotor speed deviation.

### Voltage regulation loop for steady-state recovery

In addition to inertia and damping emulation, a voltage regulation loop is used to ensure that the DC-link voltage converges to $V_{\textrm{dc}}^\star$ in steady state. This loop is typically implemented as a proportional-integral (PI) controller acting on $\Delta V_{\textrm{dc}}(t)$. The power contribution of this loop, denoted $\Delta P_{\textrm{PI}}$, is given in the Laplace domain by Yang et al.^9^

16 $$\begin{aligned} \Delta P_{\textrm{PI}}(s) = - \left( P_v + \frac{I_v}{s} \right) \Delta V_{\textrm{dc}}(s), \end{aligned}$$

where $P_v$ and $I_v$ are the primary and secondary voltage support gains. The proportional term $P_v \Delta V_{\textrm{dc}}$ provides fast disturbance rejection, and the integral term $\frac{I_v}{s} \Delta V_{\textrm{dc}}$ guarantees zero steady-state error by slowly restoring the voltage to its nominal reference.

The total commanded output power of the VSG-based controller can thus be expressed as

17 $$\begin{aligned} {\begin{matrix} P_{\textrm{cmd}}(s) & = P_{\textrm{ref}}(s) + \Delta P_{\textrm{PI}}(s) \\ & = \left( J_v s + D_v \right) \Delta V_{\textrm{dc}}(s) - \left( P_v + \frac{I_v}{s} \right) \Delta V_{\textrm{dc}}(s) \end{matrix}} \end{aligned}$$

After appropriate scaling by the measured DC bus voltage, the signal in (17) is converted into a reference current for the bidirectional DC/DC converter that interfaces the BESS with the DC bus. The inner current-control loop of the converter enforces this current reference through pulse-width modulation of the converter switches.

### Limitations of the conventional VSG

The control law in (17) improves DC voltage support compared to a basic PI controller. However, two key limitations remain: (1) The virtual inertia term $J_v s \Delta V_{\textrm{dc}}(s)$ relies on an integer-order derivative of the DC-link voltage deviation. This derivative is sensitive to high-frequency noise and may induce high-frequency oscillations in the commanded current. (2) The parameters $J_v$ and $D_v$ are constant and of integer order, which restricts tuning flexibility. In particular, there is limited capability to independently adjust the damping ratio, settling time, and overshoot of the DC-link voltage under a wide range of operating conditions, such as sudden load steps or rapid fluctuations of renewable generation. These limitations motivate the introduction of a fractional-order extension of the VSG concept, as described in the next Section.

## Proposed fractional-order virtual synchronous generator (FOVSG)

**Fig. 7:**
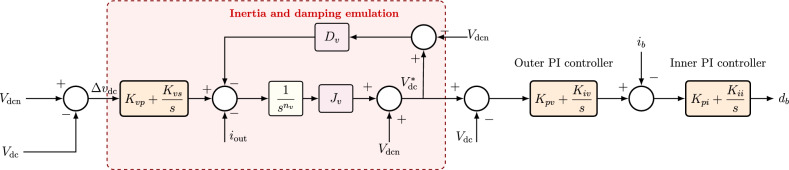
Block diagram of the proposed FOVSG for DC microgrid.

The proposed fractional-order virtual synchronous generator (FOVSG) extends the conventional VSG by introducing fractional-order dynamics into the inertia emulation path, as shown in Fig. 7. The objective is to regulate both the DC-link voltage magnitude and the rate-of-change of the DC-link voltage (RoCoV) in a smooth and robust manner, while retaining the desirable steady-state properties of conventional VSG control.

### Fractional-order operator definition

Fractional-order (FO) operators generalize differentiation and integration to non-integer orders. Let $q \in \mathbb {R}^+$ and $n \in \mathbb {N}$ be such that $n-1< q < n$. The Riemann–Liouville fractional derivative of order $q$ of a function $f(t)$ is defined as^41,42^

18 $$\begin{aligned} {\begin{matrix} D_{l_b}^q f(t) & = \frac{1}{\Gamma (n-q)} \left( \frac{d}{dt}\right) ^{\!n} \int _{l_b}^{t} \frac{f(\tau )}{(t-\tau )^{\,q-n+1}}\, d\tau ,\\ & \quad n-1<q<n . \end{matrix}} \end{aligned}$$

where $\Gamma (\cdot )$ denotes the Gamma function and $l_b$ is the lower limit of the operator. The presence of the convolution-like integral in (18) indicates that the fractional derivative possesses memory of past values of $f(t)$, in contrast to an integer-order derivative, which depends only on the instantaneous slope.

Taking the Laplace transform of (18) for zero initial conditions yields^41,42^

19 $$\begin{aligned} \mathcal {L}\left\{ D_{0^+}^q f(t) \right\} = s^q F(s), \qquad F(s) = \mathcal {L}\{ f(t) \}. \end{aligned}$$

Hence, in the frequency domain, the operator $D_{0^+}^q$ behaves as multiplication by $s^q$. For $q=1$, the usual first derivative is recovered; for $0<q<1$, a “fractional” differentiator is obtained, which responds to both present and historical deviations, providing a smoother dynamic response.

Fractional-order control structures such as the fractional-order proportional-integral-derivative (FOPID) controller exploit this property. The transfer function of a FOPID controller is

20 $$\begin{aligned} G_c(s) = K_P + \frac{K_I}{s^\lambda } + K_D s^\mu , \qquad 0 < \lambda , \mu \le 1 , \end{aligned}$$

where $K_P$, $K_I$, and $K_D$ are proportional, integral, and derivative gains, and $\lambda$, $\mu$ are the fractional integration and differentiation orders. The parameters $\lambda$ and $\mu$ increase controller tuning flexibility beyond that of standard integer-order PID controllers^43^. This additional flexibility is of particular relevance to systems dominated by power electronic converters, including islanded and weakly supported DC microgrids^42–45^.

### Fractional-order inertia and damping emulation

In a low-inertia microgrid, the primary dynamic quantity of concern on the DC side is the rate-of-change of the DC-link voltage, sometimes referred to as RoCoV:

21 $$\begin{aligned} \text {RoCoV}(t) = \frac{d V_{\textrm{dc}}(t)}{dt}. \end{aligned}$$

Large RoCoV is undesirable, because it implies abrupt voltage excursions that can overstress converters and sensitive DC loads.

To explicitly shape RoCoV, the conventional inertia term $J_v s \Delta V_{\textrm{dc}}(s)$ in (15) is generalized to a fractional-order operator,

22 $$\begin{aligned} \Delta P_{\textrm{VIC}}(s) = J_v \, s^{n_v} \, \Delta V_{\textrm{dc}}(s), \end{aligned}$$

where $0 < n_v \le 1$ is the fractional order. For $n_v = 1$, (22) reduces to a conventional first-derivative inertia term. For $0< n_v < 1$, (22) produces a generalized inertia that reacts to voltage deviations with a non-integer-rate dynamic, resulting in smoother voltage evolution and reduced high-frequency noise amplification.

Fractional damping is provided in parallel by a proportional term $D_v \Delta V_{\textrm{dc}}(s)$, which attenuates oscillations and drives the system to a new equilibrium.

### Proposed FOVSG control law

The proposed control law for the FOVSG is constructed by combining voltage support, fractional-order inertia, and damping in a cascaded form. The resulting commanded power deviation is

23 $$\begin{aligned} \Delta P_{\textrm{VSG}}(s) = - \Delta V_{\textrm{dc}}(s) \left( P_v + \frac{I_v}{s} \right) \left( J_v s^{n_v} + D_v \right) , \end{aligned}$$

where,$P_v$ and $I_v$ are the primary and secondary voltage regulation gains; $J_v$ is the generalized (virtual) inertia coefficient; $D_v$ is the generalized (virtual) damping coefficient; $n_v$ is the fractional order of the inertia path, with $0 < n_v \le 1$.

The inner bracket $\left( P_v + \frac{I_v}{s} \right)$ in (23) provides proportional-integral regulation of $\Delta V_{\textrm{dc}}(s)$. The proportional term $P_v$ produces an immediate corrective action when the DC-link voltage deviates from its nominal value, and the integral term $\frac{I_v}{s}$ guarantees that the steady-state DC-link voltage converges back to $V_{\textrm{dc}}^\star$.

The second bracket $\left( J_v s^{n_v} + D_v \right)$ in (23) imposes dynamic shaping on the corrective power command. The element $J_v s^{n_v}$ acts as an inertia emulator with adjustable order $n_v$. This term limits the instantaneous RoCoV by resisting rapid voltage changes but does so in a manner that is less aggressive and less noise-sensitive than an ideal first-order differentiator. The term $D_v$ contributes a damping action that dissipates oscillations in the DC-link voltage and reduces overshoot and undershoot following a disturbance.

The cascaded structure in (23) implies that the effective closed-loop transfer function from $\Delta V_{\textrm{dc}}$ to $\Delta P_{\textrm{VSG}}$ is not simply first order in $s$. Instead, it includes the multiplication of a PI element and a fractional-order inertial element. Consequently, the response of the BESS converter to an abrupt change in load demand or renewable injection is governed by a mixed integer/fractional dynamical operator, whose equivalent order lies between first and second order. This provides additional freedom to simultaneously minimize overshoot, settling time, and RoCoV.

For clarity, (23) can be rewritten as

24 $$\begin{aligned} \Delta P_{\textrm{VSG}}(s) = - \left[ P_v J_v s^{n_v} + P_v D_v + \frac{I_v J_v}{s^{1-n_v}} + \frac{I_v D_v}{s} \right] \Delta V_{\textrm{dc}}(s). \end{aligned}$$

Equation (24) reveals four distinct dynamic channels acting on $\Delta V_{\textrm{dc}}$:

1. $P_v J_v s^{n_v}$, a fractional-order differentiating path for fast disturbance rejection,
2. $P_v D_v$, a proportional (static) droop-like term,
3. $\dfrac{I_v J_v}{s^{1-n_v}}$, a fractional-order integrating path that accumulates voltage deviations with memory depth governed by $1-n_v$,
4. $\dfrac{I_v D_v}{s}$, an integer-order integral path that guarantees steady-state restoration.

For $n_v = 1$, the term $\frac{I_v J_v}{s^{1-n_v}}$ becomes $\frac{I_v J_v}{s^{0}} = I_v J_v$, recovering an integer-order structure. For $0<n_v<1$, a fractional-order integrator $1/s^{1-n_v}$ appears. This introduces a tunable “long memory” effect that improves robustness to repeated or slowly varying disturbances.

The commanded power $\Delta P_{\textrm{VSG}}(s)$ is then translated into a converter current reference by dividing by the measured DC bus voltage. Assuming that the BESS converter injects current $i_{\textrm{out}}(t)$ into the DC bus of voltage $V_{\textrm{dc}}(t)$, the incremental current reference associated with (23) may be written as

25 $$\begin{aligned} \Delta I_{\textrm{ref}}(s) = \frac{\Delta P_{\textrm{VSG}}(s)}{V_{\textrm{dc}}(s)} . \end{aligned}$$

The internal current control loop of the BESS converter tracks $\Delta I_{\textrm{ref}}(s)$ through fast switching control. As a result, the BESS behaves as a controllable inertia-damping device for the DC bus.

### Closed-loop DC-link voltage dynamics

The DC-link voltage is supported by the BESS converter through the capacitor dynamics of the DC bus. Let $C_{\textrm{dc}}$ denote the equivalent capacitance seen at the DC-link. The DC bus voltage dynamics satisfy

26 $$\begin{aligned} C_{\textrm{dc}} \frac{d V_{\textrm{dc}}(t)}{dt} = i_{\textrm{out}}(t) - i_{\textrm{load}}(t) + i_{\textrm{RES}}(t), \end{aligned}$$

where $i_{\textrm{out}}(t)$ is the BESS converter current injected into the DC bus, $i_{\textrm{load}}(t)$ is the total load current, and $i_{\textrm{RES}}(t)$ is the current injected by renewable sources such as photovoltaic and wind units.

Linearizing (26) around an operating point and taking the Laplace transform gives

27 $$\begin{aligned} C_{\textrm{dc}} s \, \Delta V_{\textrm{dc}}(s) = \Delta I_{\textrm{out}}(s) - \Delta I_{\textrm{load}}(s) + \Delta I_{\textrm{RES}}(s). \end{aligned}$$

The objective of the FOVSG controller is to generate $\Delta I_{\textrm{out}}(s)$ such that $\Delta V_{\textrm{dc}}(s)$ remains bounded and quickly returns to zero following disturbances $\Delta I_{\textrm{load}}$ and $\Delta I_{\textrm{RES}}$. Using (25) and (24), and assuming sufficiently fast inner current control such that $\Delta I_{\textrm{out}}(s) \approx \Delta I_{\textrm{ref}}(s)$, the closed-loop dynamics (27) acquire terms in $s^{n_v}$, $1/s^{1-n_v}$, and $1/s$. As a consequence, the characteristic equation of the overall system becomes fractional-order. This fractional-order characteristic equation allows shaping the transient poles in a higher-dimensional design space, enabling simultaneous control of overshoot, settling time, RoCoV, and steady-state error in a manner that is not achievable with integer-order VSG alone.

### Relation to virtual inertia-damping control

The structure in (23) generalizes conventional virtual inertia-damping control laws. For reference, consider the analogy with the mechanical swing equation written in torque form:

28 $$\begin{aligned} T_m - T_e = J \frac{d \omega }{dt} + D \left( \omega - \omega _n \right) , \end{aligned}$$

where $T_m$ and $T_e$ are mechanical input and electromagnetic output torque, $\omega$ is the rotor speed, $\omega _n$ is the nominal speed, $J$ is inertia, and $D$ is damping.

In a DC microgrid, the DC-link voltage is the main state reflecting instantaneous power imbalance; therefore, shaping its rate of change provides the DC-side counterpart of inertial response. A DC-side analogue can be obtained by relating current to torque and voltage to angular speed. This leads to the control expression

29 $$\begin{aligned} i_{\textrm{set}}(t) - i_{\textrm{out}}(t) = C_v \frac{d V_{\textrm{dc}}^*(t)}{dt} + D_v \left( V_{\textrm{dc}}^*(t) - V_{\textrm{dcn}} \right) , \end{aligned}$$

where $i_{\textrm{set}}$ is the reference current, $i_{\textrm{out}}$ is the measured converter current, $V_{\textrm{dc}}^*(t)$ is an internally compensated DC voltage reference, $V_{\textrm{dcn}}$ is the nominal DC bus voltage, and $C_v$, $D_v$ are virtual inertia and damping coefficients, respectively.

Under linearization, (29) yields a second-order voltage dynamic of the form

30 $$\begin{aligned} C_v \frac{d^2 V_{\textrm{dc}}^*}{dt^2} + D_v \frac{d V_{\textrm{dc}}^*}{dt} + \frac{1}{R_d} \left( V_{\textrm{dc}}^*- V_{\textrm{dcn}} \right) = \frac{d i_{\textrm{out}}}{dt}, \end{aligned}$$

where $R_d$ is the effective droop resistance and $C_v/D_v$ defines a dominant time constant. Equation (30) represents an integer-order inertial response in the DC-voltage domain. The FOVSG extends (30) by replacing the pure derivative/inertia term with a fractional-order operator, which introduces a nonlocal, memory-dependent response and thus a continuum of effective inertia behaviors parameterized by $n_v$.

### Controller tuning via metaheuristic optimization

The control law in (23) includes several parameters that strongly influence system performance:

$$\Theta = \left\{ P_v,\, I_v,\, J_v,\, D_v,\, n_v \right\} .$$

Appropriate selection of $\Theta$ is essential to guarantee fast transient recovery, suppression of oscillations, and acceptable steady-state accuracy. Manual tuning by trial-and-error is not systematic and may be challenging when the operating conditions of the DC microgrid vary significantly.

To address this issue, a metaheuristic optimization procedure based on the Grey Wolf Optimizer (GWO) is employed. GWO was selected for parameter tuning because of its low implementation complexity, limited number of algorithm-specific parameters, and effective search capability in nonlinear optimization problems, making it suitable for offline tuning of the proposed controller. The population size is set to 20 search agents, and the optimization is allowed to proceed for approximately 25 iterations. The optimization objective is defined in terms of the integral of squared error (ISE) of the DC-link voltage during representative disturbance scenarios:

31 $$\begin{aligned} \textrm{ISE} = \int _{0}^{t_{\textrm{sim}}} \left( V_{\textrm{dc,ref}}(t) - V_{\textrm{dc}}(t) \right) ^2 \, dt , \end{aligned}$$

where $t_{\textrm{sim}}$ denotes the simulation time horizon, $V_{\textrm{dc,ref}}$ is the desired DC-link voltage reference, and $V_{\textrm{dc}}$ is the actual DC-link voltage response under disturbance.

## Results and discussion

To evaluate the performance of the proposed control scheme, a simulation study of the DC microgrid (shown in Fig. 1) was conducted in the MATLAB/Simulink environment. The system parameters and component specifications used in the simulation are summarized in Table 2, with the DC microgrid’s maximum rating set at 15 kW, while the setpoint of the DC link voltage is 700 V.

**Table 2:** Utilized control parameters.

Parameter	Symbol	Value
Nominal DC bus voltage	$V_{\textrm{dcn}}$	700V
Virtual primary control gain	$k_{vp}$	8.15
Virtual secondary control gain	$k_{vs}$	65.57
Fractional-order virtual damping gain	$D_v$	4.39
Fractional-order virtual inertia gain	$J_v$	75.12
Fractional-order inertia order	$n_v$	0.42
Outer-loop proportional gain	$k_{pv}$	1
Outer-loop integral gain	$k_{iv}$	10
Inner-loop proportional gain	$k_{pi}$	0.01
Inner-loop integral gain	$k_{ii}$	5

### Case I: Load change while RESs are unchanged

**Fig. 8:**
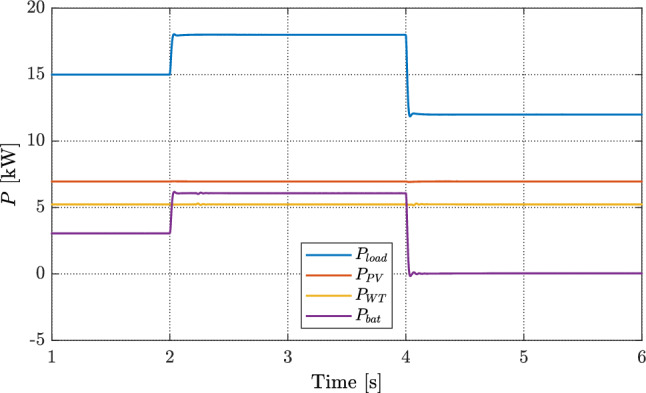
Power profile of the DGs and load with the proposed FOVSG control.

**Fig. 9:**
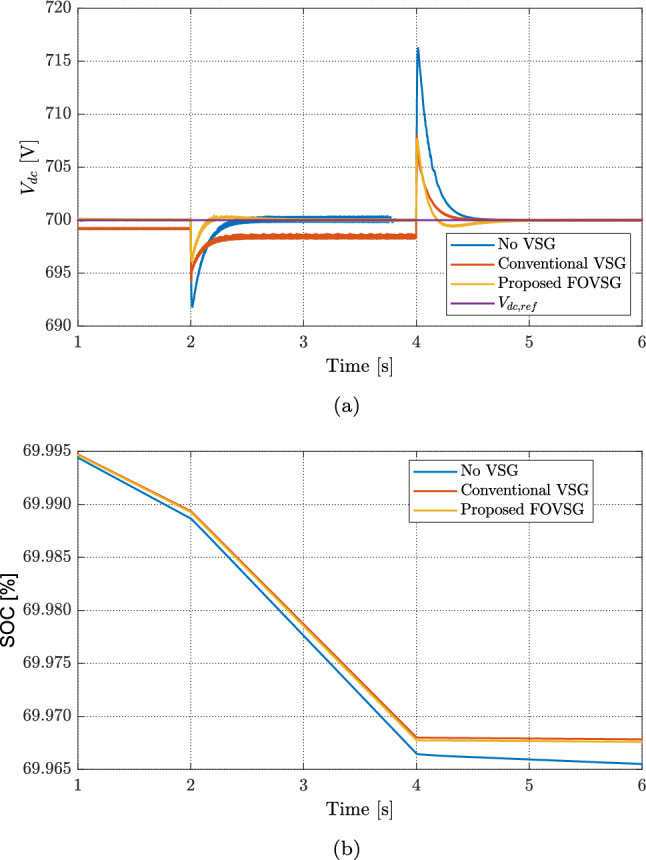
Comparison between conventional controllers and the proposed full VSG controller: (**a**) DC microgrid voltage and (**b**) battery SOC.

Under this test, a step change is applied to the load while the PV and WT outputs are kept constant; the battery compensates the power mismatch to satisfy the demand, as shown in Fig. 8. When the load varies and the RES power is fixed. Figure 9a compares the DC-link voltage response for three cases. At the load steps at $t=2$ s and $t=4$ s, the case without VSG exhibits large voltage transients of approximately 7.5 V and 16 V, respectively. With a conventional VSG, the transient is reduced but about 2 V steady-state error persists. In contrast, the proposed FOVSG further attenuates the transients to about 5 V and 7 V at the two step instants and fully eliminates the steady-state error, yielding superior regulation and disturbance rejection.

The corresponding battery behavior is reported in Fig. 9b. During the load increase (15 kW $\rightarrow$ 17 kW) at $t=2$ s, the battery discharges to supply the deficit, and the SOC drops from $69.99\%$ until the load returns to 15 kW at $t=4$ s. After the load settles, the SOC stabilizes at $69.968\%$ for both the conventional VSG and the proposed FOVSG, whereas it continues to drift downward to $69.964\%$ without VSG action. These results confirm that FOVSG improves voltage regulation without incurring additional steady-state battery depletion. Overall, the proposed strategy achieves: (i) small, well-damped voltage transients; (ii) accurate voltage tracking with no steady-state error; and (iii) minimal SOC impact, highlighting its suitability for high-quality DC-bus regulation under load variability.

### Case II: Load unchanged while RESs are changed

In this scenario, the load is fixed at 15 kW while the renewable sources change to mimic normal weather variations. The solar irradiance drops from about 1.0 to 0.75 kW/m$^2$ at $t=3$ s, similar to a quick cloud passing over the panels. The wind speed first dips from 10 to 9 m/s at $t=2$ s, then rises to 11 m/s at $t=5$ s. In the model, MPPT controllers convert these changes in sunlight and wind into corresponding changes in PV and WT power. With the proposed FOVSG, the battery makes the difference so that the DC bus stays close to its reference. This test includes decreases and increases in the renewable inputs, at different times, so we can clearly see how well the controller smooths the transients, keeps the voltage on target, and limits unnecessary battery use.

**Fig. 10:**
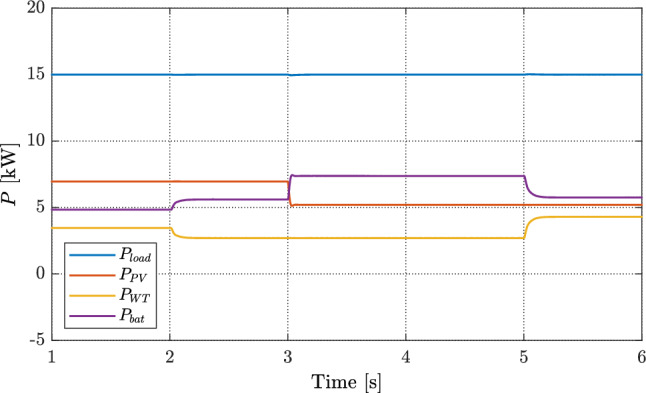
Power profile of the DGs and load with the proposed FOVSG control.

Figure 10 shows the power flows under the proposed FOVSG with a fixed load of 15 kW. The renewable sources change as: the wind speed dip at $t=2$ s causes the WT power to drop, and the irradiance step at $t=3$ s reduces the PV power. In both cases, the battery immediately supplies the missing power so that the total generation continues to match the load. When the wind increases at $t=5$ s, the WT briefly produces surplus power; the battery then absorbs this excess (charging) and returns close to zero once the new steady state is reached. Across all steps, the battery response is smooth and well damped, with no noticeable oscillations or saturation. PV and WT traces follow their resource changes while the load remains flat, confirming that FOVSG keeps the DC bus regulated by shifting only the battery channel. This demonstrates tight power balancing, quick recovery after disturbances, and minimal battery cycling outside the transient intervals.

**Fig. 11:**
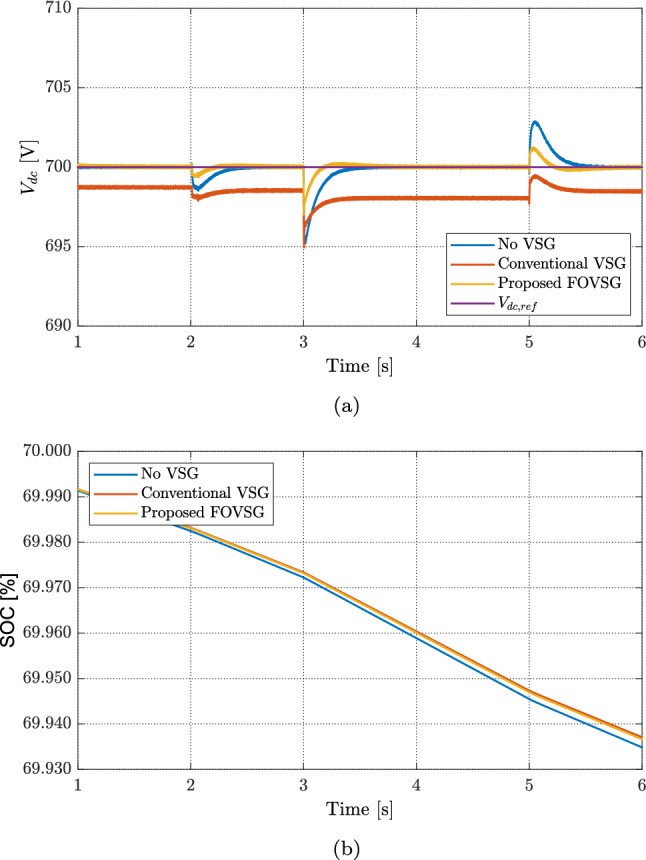
Comparison between conventional controllers and the proposed full VSG controller: (**a**) DC microgrid voltage and (**b**) battery SOC.

Figure 11 compares the DC-bus voltage and battery SOC for three controllers. In Fig. 11a, the case without VSG shows clear voltage spikes of about 2 V at $t=2$ s, 5 V at $t=3$ s, and 3 V at $t=5$ s. With a conventional VSG, the voltage tracks better but keeps a steady offset: roughly 1 V from 1—2s, 2 V from 2—3s, and 3 V from 3—5s. It also shows small extra spikes of about 3 V at $t=3$s and 2 V at $t=5$s. The proposed FOVSG removes the steady offset and further reduces the spikes to about 2 V at $t=3$s and 1 V at $t=5$s while returning quickly to the 700 V reference. This indicates stronger damping, tighter regulation, and better disturbance rejection.

The SOC trends in Fig. 11b match these behaviors. The battery discharges slowly from 2 to 3 s as the RES power is lower. After the irradiance drop at $t=3$ s, the discharge rate increases and the SOC falls from about $69.97\%$ to $69.95\%$ by $t=5$ s. Once the wind rises at $t=5$ s, the SOC stabilizes under the proposed FOVSG and the conventional VSG, while it drifts when no VSG is used. Overall, the proposed FOVSG keeps the DC voltage on target with smaller transients and avoids long-term SOC drift, showing better coordination between voltage control and battery use.

### Case III: Load change while RESs are changed

**Fig. 12:**
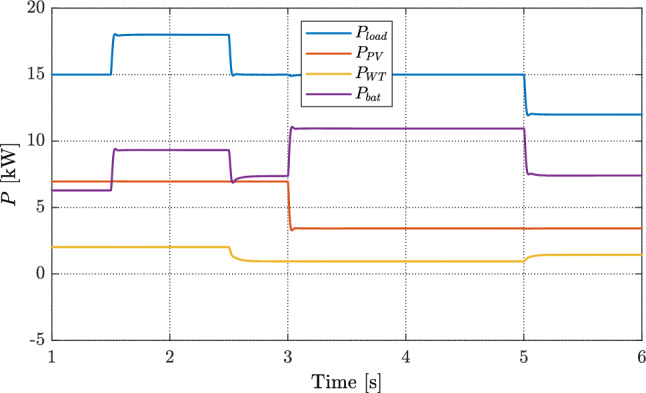
Power profile of the DGs and load with the proposed full VSG control.

In this case, the load and the renewable inputs vary at the same time. The solar irradiance drops from about 1.0 to 0.5 kW/m$^2$ at $t=3$ s; the wind speed steps from 8 to 6 m/s at $t=2.5$ s and then rises to 7 m/s at $t=5$ s. Figure 12 reports the resulting power sharing under the proposed FOVSG. The load follows a four–level profile: 15 kW from 1–1.5 s, 17 kW from 1.5–2.5 s, back to 15 kW from 2.5–5 s, and 12 kW after $t=5$ s. The RES outputs change with their resources: the PV power steps from 6 kW to 4 kW at $t=3$ s; the WT power drops from 2 kW to 1 kW at $t=2.5$ s and increases to 2 kW at $t=5$ s. The battery automatically covers the mismatch caused by these changes. It discharges during the load increase and during the PV/WT drops, and charges when the load decreases or the WT rises at $t=5$ s. Across all steps, its response is smooth and quickly settles, with no noticeable oscillations or saturation. This shows that the proposed FOVSG redistributes power cleanly between sources, keeps the RES operating points undisturbed, and uses the battery only as needed, supporting tight DC-bus regulation even when demand and renewable inputs vary.

**Fig. 13:**
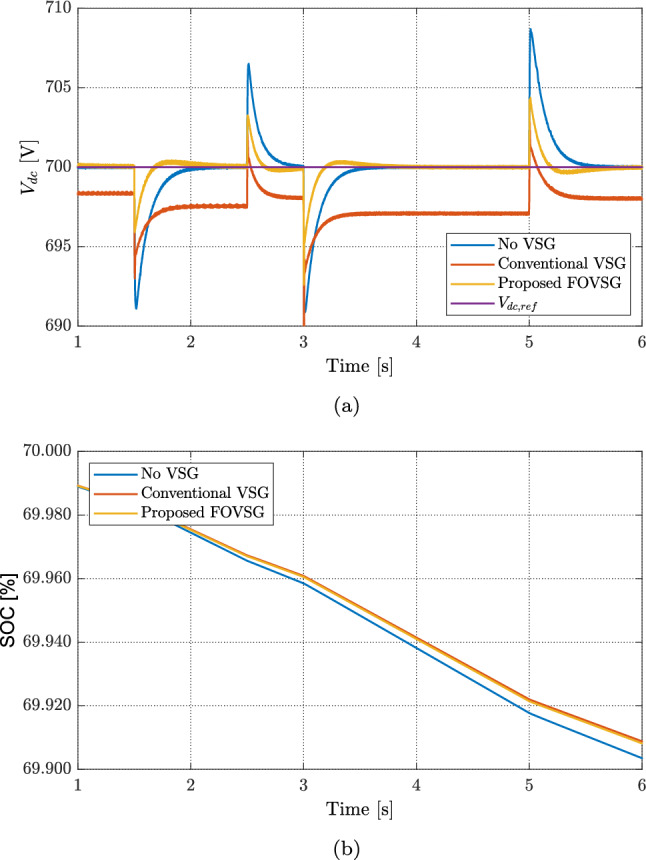
Comparison between conventional controllers and the proposed full VSG controller: (**a**) DC microgrid voltage and (**b**) battery SOC.

Figure 13 summarizes voltage regulation and battery behavior for the three controllers in one view. In Fig. 13a, without VSG the DC bus shows large spikes of about 9 V at $t=1.5$ s, 6 V at $t=2.5$ s, 9 V at $t=3$ s, and 9 V at $t=5$ s. With a conventional VSG the response is steadier but keeps a steady offset of roughly 3.5 V and reaches a maximum spike near 7 V. The proposed FOVSG removes the steady offset, limits the peak to about 6 V, and settles faster with less ringing, giving tighter voltage control when both the load and the RES powers change. The SOC traces in Fig. 13b follow the net power mismatch: between $t=3$ and $t=5$ s the battery discharges smoothly and stays within $69.960\%$–$69.920\%$ for both FOVSG and the conventional VSG, while in the no-VSG case the SOC continues to drift after the step at $t=5$ s. Overall, FOVSG coordinates the battery more effectively, reduces voltage spikes, and avoids unnecessary cycling.

### Numerical analysis

Table 3 compares the DC-link voltage transients for the three controllers across the three test cases. Overall, the proposed FOVSG consistently lowers the peak deviations while keeping settling times competitive. In Case 1, overshoot/undershoot fall from 16/7.5 V (no VSG) to 8.5/4 V with FOVSG, while the conventional VSG has smaller peaks than no VSG but introduces a noticeable steady offset (seen in the figures). In Case 2, FOVSG gives the smallest peaks (1/3 V) and matches the fastest settling time (0.3 s), improving on the no-VSG values (3/5 V, 0.7 s) and removing the offset observed with conventional VSG. In Case 3, FOVSG equals the best overshoot (4 V) and reduces undershoot to 7.5 V compared with 9 V for the other two, with a settling time comparable to the no-VSG case (0.6 s) but slightly slower than conventional VSG (0.4 s). Taken together, the results show that FOVSG achieves smaller voltage excursions in all cases and competitive settling, delivering steadier DC-link regulation without the steady-state error seen with the conventional VSG.

**Table 3:** Performance of the DC link voltage with different controllers.

Case	Metric	Conventional dual-loop without VSG	Conventional dual-loop with VSG	Proposed full FOVSG
Case 1	Overshoot (V)	16	9	8.5
	Undershoot (V)	7.5	5.5	4
	Settling time (s)	0.6	0.4	0.7
Case 2	Overshoot (V)	3	1.5	1
	Undershoot (V)	5	4	3
	Settling time (s)	0.7	0.3	0.3
Case 3	Overshoot (V)	9	4	4
	Undershoot (V)	9	9	7.5
	Settling time (s)	0.6	0.4	0.6

## Conclusion

High-penetration renewable energy sources and limited dispatchable generation in isolated DC microgrids have led to a significant reduction in system inertia, increasing their vulnerability to instability. To solve this issue, virtual inertia control has emerged as an attractive option for increasing DC link voltage stability. In this regard, a unique fractional-order virtual synchronous generator model is suggested, aiming to simulate the dynamic behaviour of a synchronous generator in DC microgrids. By incorporating fractional-order derivatives, the FOVSG introduces additional tuning flexibility, enabling superior control over system dynamics. Simulation results show that the FOVSG control scheme outperforms standard VSG methods regarding stability, with faster settling times and much lower DC link voltage variations under various operating conditions and disturbances. Specifically, the FOVSG outperforms full-order derivative-based VSG schemes by around 80$\%$ and surpasses integer-order VSG models by approximately 45$\%$ in managing load and renewable fluctuations, demonstrating its effectiveness and adaptability in diverse microgrid scenarios. The proposed FOVSG concept can be expanded for further study, including integration with sophisticated optimisation-based control strategies, coordinated operation with energy storage systems, and adaptation to hybrid AC/DC microgrid environments.

Future work will focus on experimental validation of the proposed controller, investigation of real-time implementation aspects of the fractional-order control law, including approximation and discretization methods, and assessment of robustness under parameter uncertainties, communication delays, and wider renewable penetration levels. In addition, the proposed FOVSG concept can be further extended through integration with advanced optimization-based control strategies, coordinated operation with energy storage systems, and adaptation to hybrid AC/DC microgrid environments.

## Data Availability

The datasets generated and/or analysed during the current study are available from the corresponding author on reasonable request.
